# Interlaminar Properties of Prepregs Reinforced with Multiwalled Carbon Nanotubes/Graphene Oxide

**DOI:** 10.3390/ma16155285

**Published:** 2023-07-27

**Authors:** Liwei Wen, Haiqing Shen, Zhuan Chen

**Affiliations:** 1 College of Material Science and Engineering, Nanjing University of Aeronautics and Astronautics, Nanjing 210016, China; 2 CETC Wuhu Diamond Aircraft Manufacture Co., Ltd., Wuhu 241000, China

**Keywords:** multiwalled carbon nanotubes (MWCNTs), graphene oxide (GO), interlayer reinforcement, epoxy resins, prepreg, carbon fibers

## Abstract

Carbon-fiber-reinforced polymer (CFRP) composites are widely used in industries such as aerospace due to their lightweight nature and high strength. However, weak interfacial bonding strength is one of the main problems of resin-based composites. In this study, a prepreg was prepared by melt mixing. By dispersing nanoreinforcement particles in the resin, the interlaminar shear strength of the CFRP was increased by approximately 23.6%. When only 0.5 wt% multiwalled carbon nanotube (MWCNT) was used for reinforcement, scanning electron microscopy (SEM) micrographs showed that cracks were hindered by the MWCNTs during propagation, causing crack deflection. At the same time, the mechanism of MWCNTs pulling out increased the energy required for crack propagation. When only 0.5 wt% graphene oxide (GO) was added, the reinforcement effect was inferior to that of using the same amount of MWCNTs. The laminar structure formed by GO and the resin matrix adhered to the carbon fiber surface, reducing the degree of destruction of the resin matrix, but its hindering effect on crack propagation was weak. When 0.5 wt% of MWCNT and GO mixture was added, the interlayer shear strength increased from 55.6 MPa in the blank group to 68.7 MPa. The laminar structure of GO provided a platform for the MWCNTs to form a mesh structure inside its matrix. At the same time, the tubular structure of the MWCNTs inhibited the stacking of GO, providing better dispersion and forming a synergistic enhancement effect.

## 1. Introduction

CFRP composites are preferred as advanced materials due to their lightweight, high-strength, and structural design capabilities [1,2,3]. They find extensive use in industrial fields, notably aerospace [4]. In the field of high-pressure hydrogen storage tanks, the utilization of CFRPs offers numerous advantages, including high modulus, strength, and lightweight properties. These qualities ensure both excellent quality and a weight that is only one-third of steel-inner-liner tanks. Consequently, CFRPs have gained widespread application in the realm of high-pressure hydrogen storage tanks [5]. However, the fundamental mechanical properties of resin-based composite materials depend heavily on the bonding strength between the fibers and the resin. Because of the weak bonding force between the reinforcing material and the resin surface [6,7,8], cracks induced by interlayer stress tend to propagate at the interface between the resin and fibers, thus causing component failure [9,10,11]. Consequently, the scientific community has devoted considerable attention to the study of interlayer reinforcement in composite materials.

The main methods for achieving interlayer reinforcement include oxidation treatment [12,13], matrix toughening [14,15], Z-pinning (a technique to insert reinforcing fibers along the Z-direction of continuous-fiber-reinforced plastics) [16], and nanoparticle treatment [17,18]. Due to the demand for improved anti-delamination properties without affecting in-plane stiffness and strength, the use of nanoreinforcement particles (such as TiO_2_/Al_2_O_3_ [19], nanosilica [20,21], and nanofiber materials such as CNTs) to modify CFRP composites has been widely studied by researchers. Among a multitude of reinforcing materials, carbon nanotubes and graphene oxide have garnered widespread attention due to their exceptional mechanical properties [22,23,24,25] By uniformly dispersing CNTs into the resin matrix, stress can be effectively transferred from the resin matrix to the CNTs when interlayer damage occurs, and additional energy is required to extract the CNTs from the resin matrix [26]. This fiber extraction mechanism can effectively enhance the material’s resistance to delamination. Additionally, when CNTs are aggregated, they form a mesh structure that inhibits crack propagation [27,28], leading to an increase in fracture toughness. The reinforcement mechanism of graphene is like that of CNTs. Compared with the smooth fracture surface of pure epoxy resin, the addition of graphene to the epoxy resin results in a rough surface due to crack deflection, pinning, and crack merging, all of which contribute to the increased energy required for failure [29,30]. As a result, many works have been carried out in the last years for improving the interlaminar mechanical properties of CFRPs with the incorporation of CNTs and graphene through the use of various techniques, which include electrophoresis, chemical vapor deposition, sizing, solvent casting, in situ polymerization, roll coating, electrospinning, and melt mixing [31].

Due to its simplicity, low cost, and high operability, the resin melt mixing process is applicable for large-scale industrial production [32]. Researchers have utilized this approach to achieve the interlaminar reinforcement of CFRP in a rapid and convenient manner by incorporating nano-enhancement particles into the resin matrix. For example, Bakis et al. [33] increased the critical stress intensity factor (K_IC_) by 20% and the critical strain energy release rate (G_IC_) by 38% by adding plasma-treated CNTs to an epoxy-resin-based prepreg. B. P. Singh et al. [34] achieved a 40% increase in tensile strength compared to a pure resin prepreg by incorporating a CNT prepreg into epoxy resin. Kang et al. [35] reported that adding 1.00 wt% GO to the epoxy resin resulted in an 80% increase in impact strength and a 98% increase in the K_IC_ value. Most of the introduced nanomaterial reinforcements are single one-dimensional materials, such as carbon nanotubes (CNTs) [36,37], or two-dimensional materials like graphene [38] and hexagonal boron nitride (hBN) [39]. However, relatively few studies have focused on the simultaneous introduction of one-dimensional and two-dimensional materials.

In this study, our objective is to enhance the interlaminar shear strength of annular components. We dispersed MWCNTs and GO in a resin solution and utilized a resin melt mixing process to fabricate MWCNT/GO-enhanced prepreg yarns. Subsequently, the Naval Ordnance Laboratory (NOL) rings were fabricated using an automated winding process, and short beam shear specimens were prepared from these NOL rings to test the interlaminar shear strength. A comparative analysis was conducted to evaluate the interlaminar enhancement effects when introducing only MWCNTs or GO, as well as the simultaneous incorporation of MWCNT/GO. The reinforcement mechanism was further investigated through microscopic morphology analysis using electron microscopy.

## 2. Materials

The T700SC-12K-50C carbon fiber used in this study was manufactured by Toray Industries Inc. (Tokyo, Japan). The sizing agent used was of type 50C, and the epoxy resin content was 1%. The carbon fibers have a smooth surface and excellent quality, making them suitable for the preparation of the prepreg. The specific parameters of T700 are shown in Table 1.

The 5212-epoxy resin composite material was sourced from Shanghai Huayi Resin Co., Ltd. (Shanghai, China) and is composed of two components, A and B, with a mass mixing ratio of 100:42 (A:B). The parameters for components A and B, as well as the 5212-epoxy resin composite material, are shown in Table 2.

Carboxylated MWCNTs and GO enhance the interfacial interaction with the polymer matrix during the formation process by forming covalent or noncovalent bonds with the epoxy resin. This interaction improves the dispersion of the nanofillers in the polymer. Therefore, in this experiment, carboxylated MWCNTs and GO were selected as the nanoreinforcement particles. The hydroxylated multiwalled carbon nanotubes (MWCNTs) were purchased from Jiazhaoye (Guangdong, China) New Materials Co., Ltd., while the graphene oxide (GO) was produced by Shenzhen Suiheng Technology Co., Ltd. (Shenzhen, China). The specific parameters of the carboxylated MWCNTs and GO are listed in Table 3.

### 2.1. Prepreg Preparation

The experiment was divided into four groups. The first group was the blank group, while the other three groups had a mixture of nanometer particles added to the resin in the following mass fractions: 0.5% MWCNTs, 0.5% GO and 0.25% MWCNTs + 0.25% GO, respectively. The MWCNT/GO/MWCNT-GO nanoparticles were poured into a beaker containing epoxy resin and mechanically stirred for 5 min to initially disperse the carbon nanotubes above the resin. Then, the mixture was transferred to a constant-temperature magnetic stirrer with a temperature setting of 30 °C and a rotor speed of 600 rpm. The rotor was magnetically stirred under high-speed conditions for 20 min (the constant-temperature magnetic stirrer was produced by Changzhou Jintan Liangyou Instrument Co., Ltd., Changzhou, China). Finally, the MWCNT/GO/MWCNT-GO epoxy resin solution after magnetic stirring was subjected to ultrasonic dispersion treatment. The temperature was set to 30 °C, the frequency was set to 20 KHz, and the power was set to 200 W. The ultrasonic dispersion was carried out for 2 h (the ultrasonic disperser was produced by Shanghai Yuming Instrument Co., Ltd., model YM-500, Shanghai, China).

**Figure 1:**
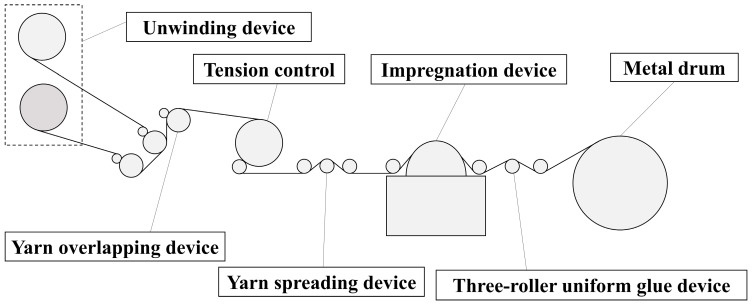
Prepreg preparation platform.

After dispersion was completed, the prepreg yarn was prepared using hot melt mixing. Two strands of yarn were merged by the yarn-merging device and then dipped into the resin. The resulting prepreg yarn had stable width and resin content. The preparation platform is shown in Figure 1. Firstly, constant tension was provided by a torque motor to rotate the unwinding device at a uniform speed. The tension was set to 8 N, and the traction speed was 5 m/min. Then, after being shaped by three U-shaped grooves in the yarn-merging device, the two strands of yarn were completely overlapped. After being spread out, they were dipped into the resin in the impregnation device at a constant temperature of 30 °C. The thickness of the resin film was controlled by the resin-wiping element in the impregnation device and was set to 0.2 mm. After being impregnated by a single-sided resin roller, the prepreg yarn on the side close to the impregnation roller had a higher resin content. By using a three-roller uniform glue device, the impregnation ability of the resin was increased, and the distribution of the resin was improved, resulting in a prepreg yarn with a uniformly distributed resin content.

### 2.2. Multifilament Tensile Test

**Figure 2:**
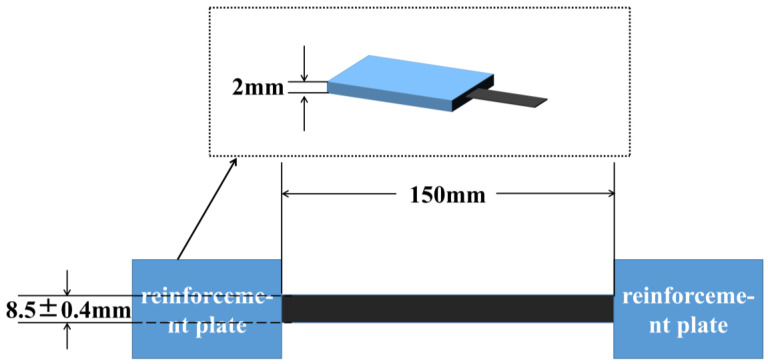
Schematic diagram of the sample dimensions and clamp spacing.

The validation of the performance of the prepreg preparation was achieved by conducting tensile tests on carbon fiber tows following the standard test method for properties of continuous-filament carbon and graphite fiber tows (ASTM D4018-11). The sample dimensions and clamp spacing are shown in Figure 2. The reinforcement patches were attached following the method shown in the experimental standard. For the patches, 1 mm thick metal plates were used, and they were bonded to the sample using AB glue. The tensile test samples were prepreg prepared using hot melt mixing, with a width of 8.5 ± 0.4 mm and a resin content of 35% by weight. The spacing between the reinforcement patches was set at 150 mm to ensure a sufficient working length. The experiment was designed to conduct tensile strength testing on a group of ten samples, using a computer-controlled electronic universal testing machine (The CMT5105 model is produced by Mettler-Toledo Industrial Systems (China) Co., Ltd., Shanghai, China). The testing machine was set to a 2 mm/min loading speed.

### 2.3. Preparation of the Short Beam Shear Sample

**Figure 3:**
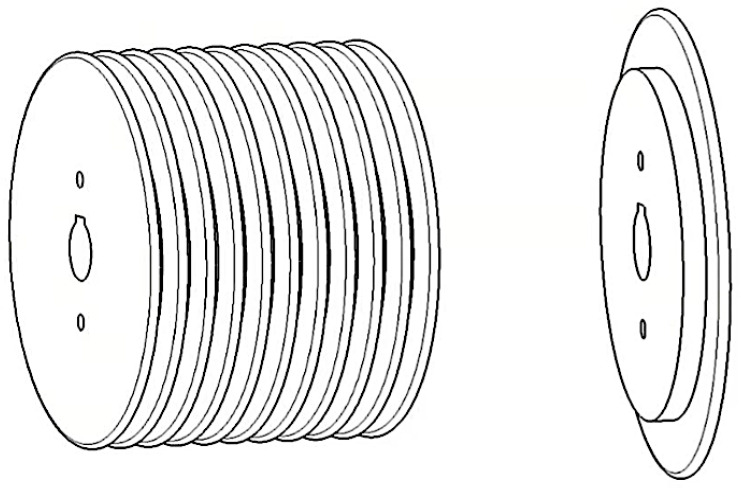
NOL ring short beam shear sample and experimental device.

To wind the NOL rings, the NOL ring mold was fixed at the rear end of the winding device using a key groove. The mold comprised NOL rings that were 9 mm in width. Each ring was connected to the adjacent ones using three bolts, and two staggered threaded holes were present on each ring. The schematic diagram of the mold is shown in Figure 3. To facilitate the demolding of the NOL rings, the mold surface was coated with a mold-release agent three times before winding. The winding tension was controlled by a DC torque motor, adjusting the winding tension to a constant 45 N by controlling the voltage. After winding, the NOL rings were subjected to a curing treatment of 120 °C for 2 h followed by 150 °C for 4 h. After the curing process was complete, the mold was allowed to cool to room temperature, and then the NOL rings were removed from the mold.

### 2.4. Testing and Characterization

**Figure 4:**
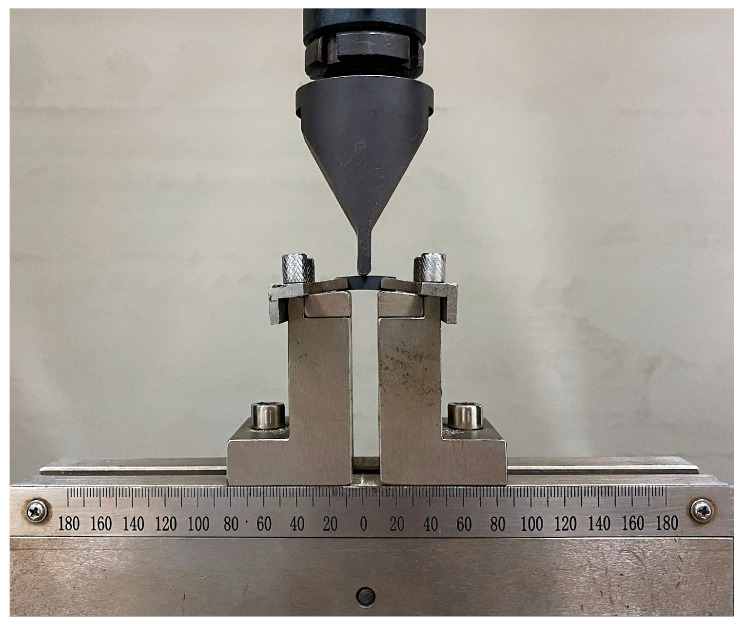
Schematic diagram of the short beam shear experiment.

The resin (blank group, 0.5%wt MWCNT group, 0.5 wt% GO group, 0.25 wt% MWCNT + 0.25 wt% GO group) was subjected to dynamic DSC testing using a differential scanning calorimeter (model DSC-200-F3, produced by Netzsch Instruments GmbH, Selb, Germany), with a heating rate of 9 °C/min from room temperature to 250 °C, to analyze the ability of MWCNT/GO to combine with the resin. To test the interlayer shear strength of the prepreg after introducing MWCNT/GO, samples were prepared according to the thickness:width:length ratio of 1:2:6 for short beam shear testing. A computer-controlled electronic universal testing machine (model CMT5105, produced by Mettler-Toledo Industrial Systems (China) Co., Ltd.) was used for the short beam shear test, with a loading rate of 2 mm/min. The short beam shear testing device is shown in Figure 4. To analyze the reinforcement mechanism of MWCNT/GO, the microstructure of the blank group and the MWCNT/GO group were observed using a Hitachi Regulus 8100 SEM (Tokyo, Japan) at an accelerating voltage of 5.0 kV and 15.0 kV.

## 3. Results and Discussion

### 3.1. The Performance of the Prepreg Prepared by Melt Mixing

The prepreg prepared by melt mixing has a width of 8.5 ± 0.4 mm and resin content of 35% by weight. The variance of the width is 0.027. The average failure load of 6 effective filament tensile samples is 3798 N, and the measured tensile strength is 4316 MPa. The theoretical tensile strength of T700 is 4900 MPa, which indicates a fiber damage rate of 11.9%. The filament tensile strength shows that the prepreg prepared by the melt mixing has a lower fiber damage rate, meeting the performance requirements of high strength and low damage for the prepreg.

### 3.2. The Ability of MWCNT/GO to Bond with the Resin

**Figure 5:**
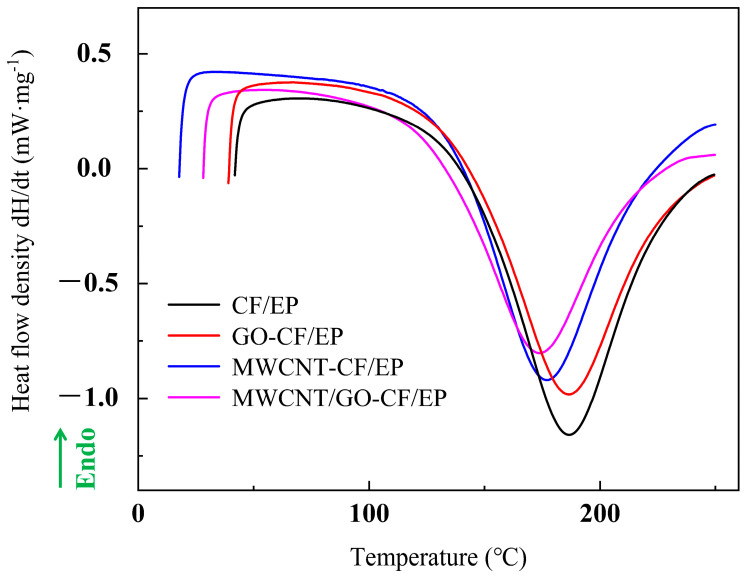
DSC curves of EP with different treatment methods.

The surface of an MWCNT contains a large number of carboxyl groups, while the surface of GO contains oxygen-containing functional groups such as carboxyl, hydroxyl, and carbonyl. The presence of these functional groups can create a good physical affinity between MWCNT/GO and the resin matrix, minimizing their tendency to aggregate. DSC curves were obtained for resin matrices with the addition of 0.5% MWCNT, 0.5% GO, and 0.25% MWCNT + 0.25% GO, respectively. As shown in Figure 5, the addition of 0.5% GO resulted in a smaller shift of the exothermic peak toward lower temperatures, while the peak temperature decreased from 186.6 °C to 176.7 °C upon the addition of 0.5% MWCNT. With the addition of 0.25% MWCNT + 0.25% GO, the peak temperature decreased from 186.6 °C to 173.7 °C. The analysis suggests that the hydroxyl and carboxyl groups present in MWCNT/GO act as effective catalysts, facilitating the curing reaction of the epoxy resin while undergoing new chemical reactions with the resin, such as cross-linking. This results in the formation of a three-dimensional cross-linked network structure. These physicochemical interactions contribute to the improvement in the microstructural toughness to a certain extent [40].

### 3.3. Interlayer Shear Strength of the Braided Composite

The short beam sample experienced failure under shear force. Experimental data revealed that the interlayer shear strength of the blank group was 55.6 MPa. The addition of 0.5% MWCNT resulted in a stronger interlayer reinforcement effect than 0.5% GO, with interlayer shear strengths of 64.98 MPa and 60.08 MPa, respectively. When the total amount is kept constant and MWCNTs are mixed with GO and added, the interlaminar shear strength is further improved, increasing to 68.7 MPa. The interlaminar shear strength and failure load of the four sample groups are shown in Table 4.

**Figure 6:**
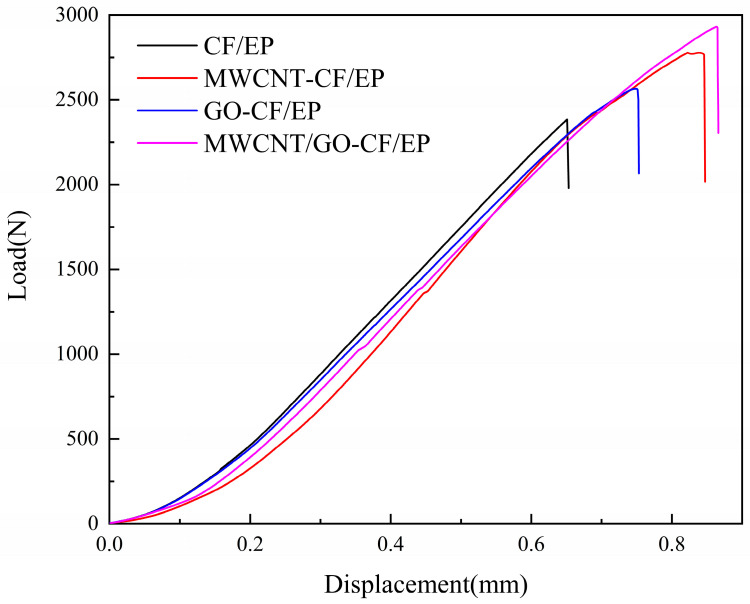
Load–displacement curves of interlaminar shear samples with different MWCNT/GO additions.

Macroscopically, interlaminar shear failure is characterized by the delamination between the samples and the presence of obvious cracks. Microscopically, the mechanism of failure is attributed to the debonding between the reinforcing carbon fibers and the resin matrix. The load–displacement curves of the NOL ring short beam shear samples with different MWCNT/GO addition levels are shown in Figure 6. When the displacement reaches the critical value, the composite material experiences delamination failure, resulting in a sharp drop in load. In the absence of MWCNT/GO, the critical failure load of the short beam sample was 2374 N. Introducing 0.5 wt% of GO increased the critical displacement from 0.65 mm to 0.75 mm, and the critical load increased from 2374 N to 2565 N, an increase of 8.1%. This is because the functional groups on the surface of oxidized graphene oxide (GO) form chemical bonds with the carbon fibers and resin matrix, thereby enhancing interfacial bonding. Moreover, the relatively large specific surface area of GO increases the bonding area between the carbon fiber reinforcement and resin matrix, allowing the load to be rapidly transferred and avoiding stress concentration. The strengthening effect was further enhanced when 0.5 wt% of MWCNT was introduced compared to 0.5 wt% of GO. The critical displacement was further increased, and the critical load increased to 2774 N, a 16.8% increase compared to the blank group. When 0.25 wt% GO and 0.25 wt% MWCNT were introduced simultaneously, the critical load was higher than when either MWCNTs or GO were added alone, increasing to 2930 N, a 23.4% increase compared to the blank group. This is because introducing nanoparticles of similar size synergistically improves the interlayer shear strength of composite materials. An observation of the sectional crack revealed that the MWCNT/GO synergistically reinforced composite material had a smaller opening crack. This indicates that when interlayer failure occurs, the load can be effectively transferred, and the energy produced by delamination can be absorbed promptly, while hindering the expansion of cracks, achieving the effect of improving interlayer mechanical properties.

### 3.4. Analysis of the Interlayer Reinforcement of Short Beam Samples with MWCNT/GO

#### 3.4.1. Fracture Surface Analysis

**Figure 7:**
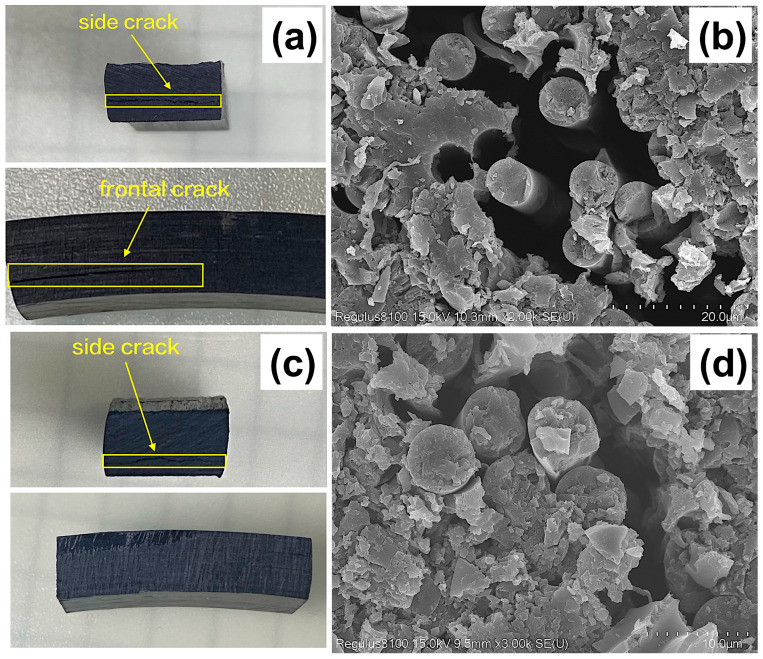
Short beam sample section cracking: (**a**) blank group sample, (**b**) side crack of the blank group, (**c**) MWCNT/GO-added group, (**d**) side crack of the MWCNT/GO-added group.

Figure 7a illustrates the occurrence of interlaminar failure in the blank group under shear force. The crack first appears on the side, then propagates to the front and continuously grows there. An SEM magnification observation reveals that the mechanism for crack formation is the debonding between the reinforcing carbon fibers and the resin matrix (Figure 7b). This indicates that the fiber–resin interface has a limited ability to suppress the continuous propagation of cracks and cannot effectively absorb the energy of cracks. A visual observation of the sample with added MWCNT/GO (Figure 7c) reveals that although cracks still exist on the side, the gaps are significantly smaller than those in the blank group. Meanwhile, the cracks do not further propagate to the front. The form of the cracks also differs from the “straight-line” shape in the blank group, presenting a “curved” appearance. Subsequently, an SEM magnification observation of the microstructure of the sample with added MWCNT/GO showed that the cracks generated under shear force were smaller compared to those in the blank group (Figure 7d), indicating that MWCNTs can enhance the binding ability between the resin matrix and the reinforcing carbon fibers to some extent and hinder the propagation of cracks.

**Figure 8:**
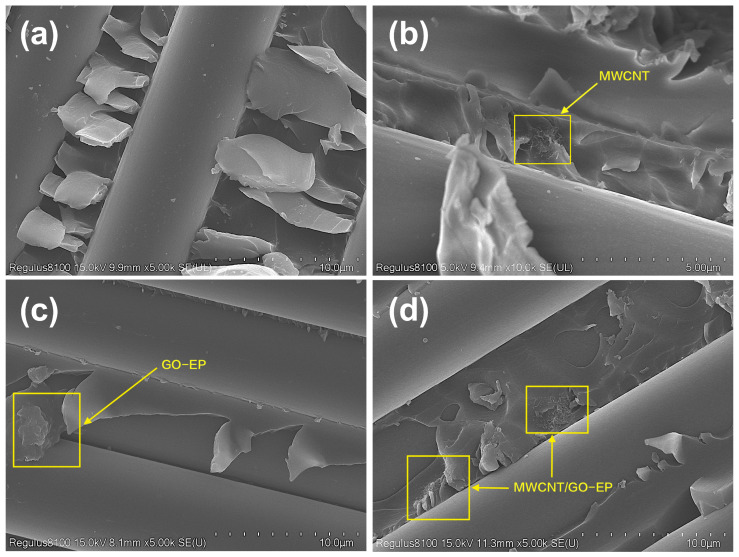
The morphology of CF/EP at different treatment sections: (**a**) CF/EP, (**b**) 0.5 wt% MWCNT-CF/EP, (**c**) 0.5 wt% GO-CF/EP, (**d**) 0.25 wt% MWCNT + 0.25 wt% GO-CF/EP.

The section that has undergone delamination damage was separated by a cutting machine, and the fracture surface of the test sample was observed using SEM. The blank group’s SEM micrograph (Figure 8a) reveals a smooth and clean surface without any fiber bridging, and the resin matrix is severely curled up. This is a typical brittle fracture feature, which proves that the resistance received by the crack during the propagation process is extremely low. The sample’s fracture surface (Figure 8b) with the 0.5 wt% MWCNT additive is relatively rough, and the adhesion between the resin and fibers is stronger. Meanwhile, there is a phenomenon of crack deflection. This indicates that the MWCNTs distributed in the resin matrix not only improve the interfacial bonding strength by forming fiber bridging but also cause crack deflection. These combined mechanisms lead to an enhancement of the interlayer shear strength. An observation of the fracture surface of the sample with the 0.5 wt% GO additive (Figure 8c) reveals a layered structure formed by the GO and resin matrix adhered to the carbon fiber surface, which leads to a weakening of the degree of destruction in the resin matrix. Meanwhile, GO aggregates in the resin matrix, but this aggregation does not hinder crack propagation or fiber bridging formation. This also confirms that the improvement in interlaminar shear strength is greater when introducing MWCNTs alone compared to introducing GO alone. The fracture surface of the sample added with MWCNT/GO composite particles (Figure 8d) exhibits a better preservation of the morphology of the fibers and resin surface, indicating that the MWCNT/GO-EP region has borne some of the destructive load. Compared with the blank group and the separate introduction of MWCNTs and GO, MWCNT/GO demonstrates a stronger interfacial bonding force between the carbon fibers and resin matrix.

**Figure 9:**
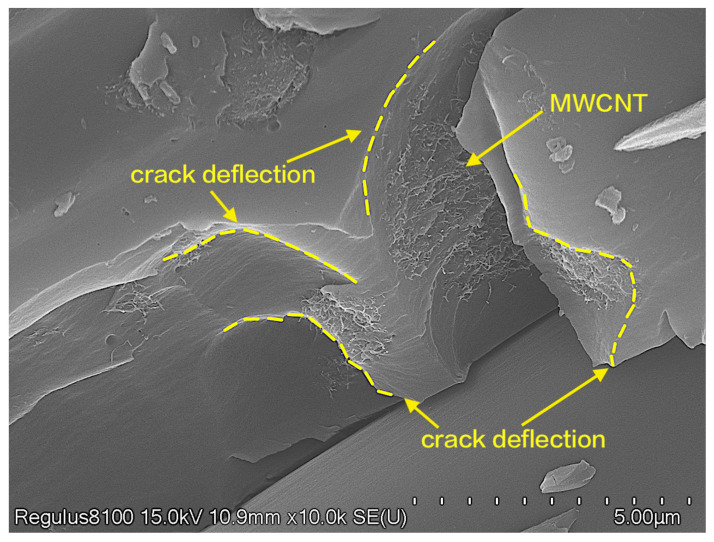
The schematic diagram of MWCNT/GO promoting crack deflection.

**Figure 10:**
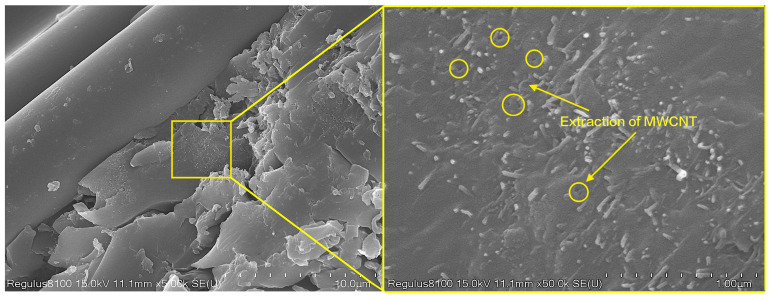
Pull-out enhancement schematic of MWCNTs.

Further enhanced analysis was conducted on the fracture surface of the short beam shear samples, as shown in Figure 9. The MWCNTs formed a mesh structure on the surface of the resin matrix, providing more adhesive points for the carbon fibers and resin matrix. In addition, MWCNTs tend to aggregate to a certain extent in the resin, which caused the crack to deflect during its propagation process, greatly increasing the total crack propagation length and thus increasing the energy required for interlaminar failure. Observations of the resin matrix rich in carbon nanotubes (Figure 10) reveal the presence of many pores on the matrix, and the MWCNTs exhibit a worm-like morphology, indicating that some of the MWCNTs were pulled out. This suggests that the addition of MWCNTs to epoxy resin can consume more energy through mechanisms such as increasing interfacial bridging resistance, improving the pull-out ability of carbon nanotubes, and increasing the interfacial debonding area, thereby enhancing interlaminar strength.

#### 3.4.2. Synergistic Enhancement Analysis

The addition of either MWCNTs or GO alone can increase the interfacial fracture toughness by forming rough surfaces and enlarging the contact area. However, SEM observations showed that when GO was introduced alone, it tended to aggregate in the resin, but this did not cause crack deflection. Therefore, the enhancement effect of GO alone was not ideal. In contrast to the sheet-like structure of GO, MWCNTs, as a one-dimensional reinforcement material, have a worm-like shape. MWCNTs form a network structure by aggregating to cause crack deflection and improve the required energy for fracture by their fiber pull-out mechanism.

**Figure 11:**
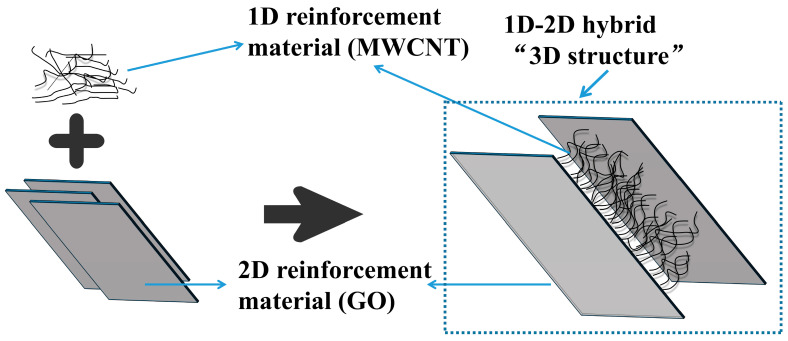
MWCNT/GO synergistic enhancement.

The experimental data show that the interlayer shear strength improvement brought by the introduction of the MWCNT/GO composite particles is greater than that of introducing MWCNTs or GO alone, indicating a synergistic reinforcement effect between the two. Analysis and speculation suggest that the sheet-like structure of GO provides a rectangular platform for the MWCNTs, which form a grid structure inside the platform [41]. In addition, a stable connection is formed between the MWCNTs and GO through van der Waals forces and interactions between the oxygen-containing functional groups. At the same time, the tubular structure of MWCNTs inhibits the stacking of GO, causing GO to distribute along the MWCNTs and form a 3D structure (Figure 11). This 3D structure provides better dispersion and results in a synergistic reinforcement effect.

## 4. Conclusions

This study employed melt mixing to prepare a prepreg with stable width and resin content. Interlayer reinforcement was achieved by introducing MWCNT/GO nanoparticles into the resin. The study compared the interlayer reinforcement effect of adding only MWCNTs or GO and adding MWCNT/GO simultaneously. The results showed that adding 0.5 wt% MWCNT increased the interlayer shear strength to 64.98 MPa, while adding 0.5 wt% GO alone resulted in a strength of 60.08 MPa, which was approximately 16.87% and 8.05% higher than the blank group. When adding 0.25 wt% MWCNT + 0.25 wt% GO, the interlayer shear strength reached 68.7 MPa, which was approximately 23.56% higher than the blank group. This indicates that the introduction of MWCNTs and GO plays a certain role in the interlayer reinforcement of the prepreg, and there is a synergistic enhancement effect when both are introduced simultaneously.

SEM was used to observe the micromorphology of the fracture surface to further analyze the reinforcement mechanism. It was found that the resin matrix rich in MWCNTs contained many pores, indicating that MWCNTs were pulled out. At the same time, MWCNT agglomeration formed a mesh structure that caused crack deflection. These mechanisms combined to increase the energy required for failure. In contrast, it was found that the strength increase was not significant when only GO was added because GO strongly agglomerates in the resin, but this aggregation does not cause crack deflection. When MWCNTs and GO were introduced simultaneously, the situation changed. GO was distributed along the mesh structure of the MWCNTs. It was analyzed that the tubular structure of MWCNTs inhibited GO stacking, enabling a better dispersion of GO and producing a synergistic enhancement effect. This study contributes to understanding the mechanism of interlayer reinforcement of MWCNTs and GO in prepregs and analyzes the synergistic reinforcement effect of MWCNTs and GO. However, due to the characteristics of the materials, there is an aggregation of MWCNTs and GO in the resin, which results in a less than ideal dispersion. Future research is expected to pave the way for nanoparticle widespread implementation in reinforced CFRP composite materials by enhancing its dispersion capability within the resin.

## Figures and Tables

**Table 1:** The specific parameters of T700.

Materials	Tow/K	Tensile Strength /MPa	Tensile Modulus /GPa	Linear Density /g·m^−1^
T700SC-12000-50C	12	4900	230	0.8

**Table 2:** The specific parameters of the 5212-epoxy resin composite material.

Materials	Specific Parameters				
5212-A	Exterior		Viscosity (mPa·S)		Epoxy value (eq/100 g)
	brownish yellow liquid		500–1500		0.83–0.93
5212-B	Exterior		Viscosity (mPa·S)		
	brownish yellow liquid		150–300		
5212- epoxy compound	Tensile strength/MPa	Tensile modulus/GPa	Bending strength/MPa	Bending modulus/GPa	Impact strength/kJ/m^2^
	80–100	3.2–3.5	145–170	3.2–3.6	25–40

**Table 3:** The specific parameters of the MWCNTs and GO.

Materials	Specific Parameters				
MWCNTs	Specific surface area m^2^/g	Bulk density g/cm^3^	Diameter/nm	Carbon nanotube legth /μm	Purity/%
	250–270	60.09	3–15	15–30	>97
GO	Layers	Single-layer diameter/μm	Thickness/nm	Exterior	Purity/%
	1–2	0.2–10	~1	brown powder	98

**Table 4:** The interlaminar shear strength and failure load of the four sample groups.

Intensity Parameter	Blank Group	0.5 wt% MWCNT Group	0.5 wt% GO Group	0.25 wt% MWCNT + 0.25 wt% GO Group
interlaminar shear strength (MPa)	55.6	64.98	60.08	68.7
failure load (N)	2374	2774	2565	2930

## Data Availability

The data that support the findings of this study are available from the corresponding authors upon reasonable request.
